# An unusual lesion of cerebellopontine angle as a pathological and diagnostic enigma: a comprehensive case report

**DOI:** 10.11604/pamj.2026.53.68.50254

**Published:** 2026-02-09

**Authors:** Nirlipta Swain, Suhit Naseri, Simran Sameer Khan, Amol Gupta

**Affiliations:** 1 Department of Pathology, Jawaharlal Nehru Medical College, Datta Meghe Institute of Higher Education and Research, Sawangi (Meghe), Wardha, Maharashtra, India,; 2 Department of Pathology, Datta Meghe Institute of Medical Sciences, Nagpur, Maharashtra, India,; 3 Department of Surgery, Jawaharlal Nehru Medical College, Datta Meghe Institute of Higher Education and Research, Sawangi (Meghe), Wardha, Maharashtra, India

**Keywords:** Peripheral nerve, neurofibromatosis, diplopia, sarcoma, case report

## Abstract

Cerebellopontine angle (CPA) tumours comprise a pathologically varied group of tumours owing to the origin of the tumour and effect on neurovascular structures, leading to a myriad of clinical manifestations. These tumours are benign, sluggish, with a low potency for malignant transformation. Malignant peripheral nerve sheath tumours (MPNST) are a malignant neoplasm of the peripheral nerves. It may develop from a pre-existing neurofibroma or may arise sporadically. Malignant peripheral nerve sheath tumours have a poor overall prognosis owing to their aggressive nature and limited therapeutic options. It comprises of 5-10% of all soft tissue sarcomas. Malignant peripheral nerve sheath tumours primarily occur in the trunk and lower extremities as opposed to intracranial location, which makes this particular site of occurrence a rarity, posing a diagnostic dilemma. We report the case of an adult male in his early 60s who presented with complaints of diplopia with headache, and a tingling sensation in the left side of his face for the past 2 months. Magnetic resonance imaging (MRI) was done, which suggested a left tentorial meningioma. Histopathological analysis revealed MPNST, which further validated the diagnosis. High mortality rates and poor prognosis stem from diagnostic dilemmas and limited treatment alternatives. This case report contributes to the existing literature by broadening the differential diagnosis of CP angle lesions and underscoring the importance of tissue diagnosis in atypical clinical presentations.

## Introduction

Malignant peripheral nerve sheath tumour (MPNST) refers to malignant tumours of peripheral nerves or nerve sheath cells apart from the epineurium [1]. Notwithstanding that these tumours typically occur over the neck, forearm, lower leg, and buttock regions, they can also arise from the cranial nerves and their branches. Most commonly involves the vestibulocochlear nerve or eighth cranial nerve [2]. Representing 5-10% of all soft tissue sarcomas, makes it a rare entity. It was in 2013 that the World Health Organization (WHO) categorized MPNST as a soft tissue sarcoma [1]. MPNSTs primarily affect the adult population, with only 10-20% of cases reported in the age group less than 20 years, with no gender preponderance. A major risk factor for MPNST is Type 1 Neurofibromatosis (NF1), which accounts for about half of all diagnosed cases. They can also be seen in post irradiation patients. Loss of neurofibromin function activates Ras kinase and its downstream pathways, promoting malignant transformation. The extent of Ras activation and cellular sensitivity to inhibitors inversely correlate with neurofibromin levels. Activated Ras triggers the Mitogen-Activated Protein Kinase (MAPK) (Ras/Raf/MEK/Erk) and Akt/mTOR pathways, which regulate cell growth and response to external signals; both are known to be upregulated in various sarcomas, including MPNST [3].

In NF1-associated MPNSTs, somatic NF1 mutations typically involve large deletions encompassing most or all of the NF1 gene, and in some cases, even the entire chromosome, except in individuals with germline microdeletions. Plexiform neurofibromas that show premalignant histologic features, referred to as “atypical neurofibromas”, frequently harbour deletions in the CDKN2A/B locus and possess an increased likelihood of progressing to MPNST [4]. The transformation to MPNST is marked by increased genetic instability, loss of Polycomb Repressive Complex 2 (PRC2) function, and amplification of genes involved in cell growth and pluripotency. In our study, the polycomb repressor complex 2 (PRC2) components Embryonic Ectoderm Development (EED) and SUZ12 were commonly found to be mutated, deleted, or reduced in expression in MPNSTs, but not in atypical neurofibromas (ANFs) [5]. It has been observed that elevated levels of Epidermal Growth Factor (EGF) receptor in Nf1-deficient peripheral nerve glial cells can drive the progression of benign neurofibromas into aggressive MPNSTs. Furthermore, activated STAT3 was notably present in MPNSTs, and treatment with a JAK2/STAT3 inhibitor was found to slow tumour growth.

Retrospective analyses of MPNST cases in individuals with NF1 have shown a 5-year disease-free survival rate ranging from 34% to 60%, which is slightly lower than that observed in sporadic MPNSTs (in individuals without NF1) [4]. The only effective treatment option for MPNSTs continues to be surgical resection with adequately wide negative margins, though it is often challenging due to tumour size, location, or metastasis. Radiotherapy aids local control, particularly for large (>5 cm), high-grade, or margin-positive tumours. Targeted noncytotoxic therapies have shown limited benefit so far, though preclinical studies suggest promise for combinatorial approaches. Notably, Tyrosine Kinase Inhibitors (TKIs) combined with MEK inhibition have demonstrated synergistic suppression of tumour growth and metastasis in MPNST xenograft models [6-8].

## Patient and observation

**Patient information:** a 60-year-old man presented to the Neurosurgery Outpatient Department with a two-month history of progressively worsening diplopia, accompanied by headaches and tingling sensations on the left side of the face. He denied any history of loss of consciousness, vomiting, or seizures. The patient had previously consulted at a private clinic with the same complaints, and he was asked to get an MRI done. MRI brain was suggestive of left tentorial meningioma, whereupon he was instructed to visit a tertiary centre for further management. The patient was a known case of diabetes and was being managed with oral hypoglycaemic drugs for the past two months, and he was diagnosed with hypertension for which he has been put on anti-hypertensives for the last eight days. He also gave a history of Double J stent placement one month back.

**Clinical findings:** the patient appeared vitally stable, and no other systemic abnormality was detected on general clinical examination.

**Timeline of current episode:** the patient gave a two-month history of progressively worsening diplopia associated with headache and tingling sensation over the left side of the face for two months.

**Figure 1:**
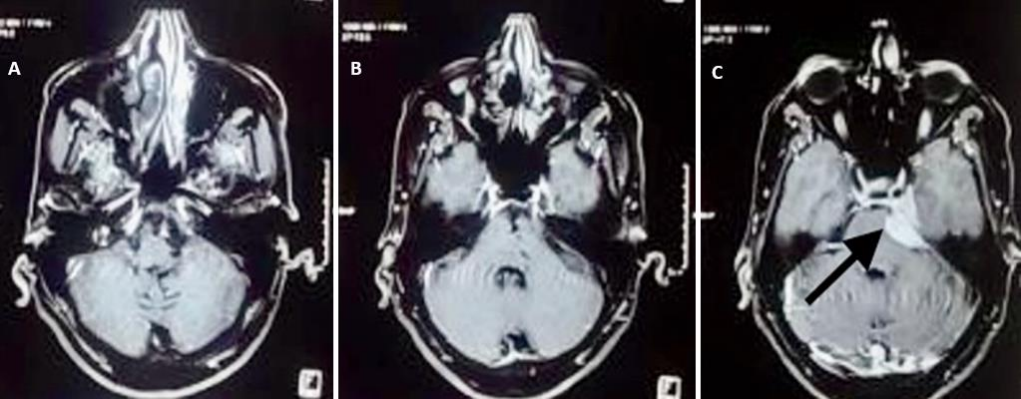
A, B, C) magnetic resonance imaging of the brain, causing post-contrast enhancement with a positive dural trail sign

**Figure 2:**
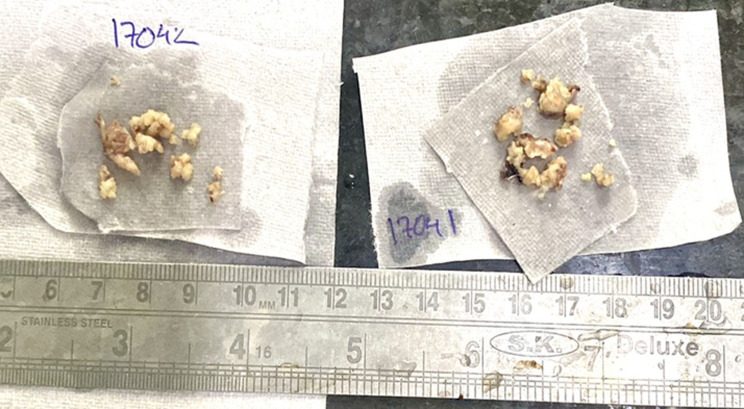
gross image of the excised specimen received in histopathology

**Figure 3:**
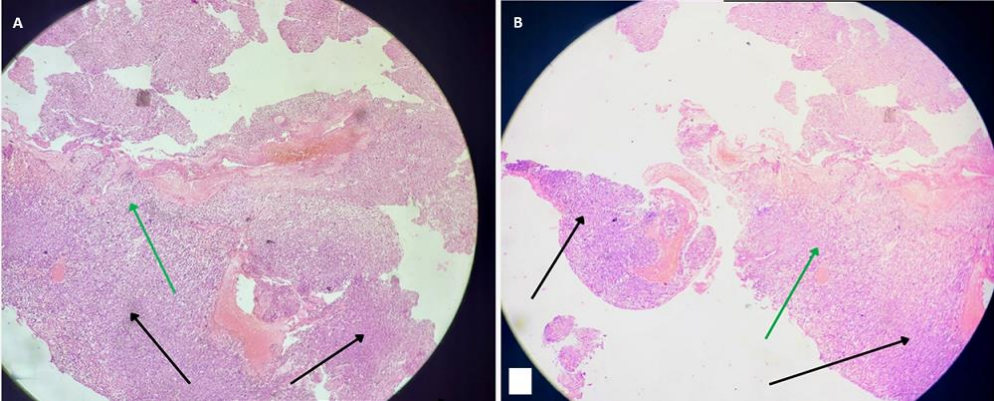
A, B) microscopic examination [H&E, 10X]- alternate hypocellular (green arrows) and hypercellular areas (black arrows) producing a marbling effect

**Figure 4:**
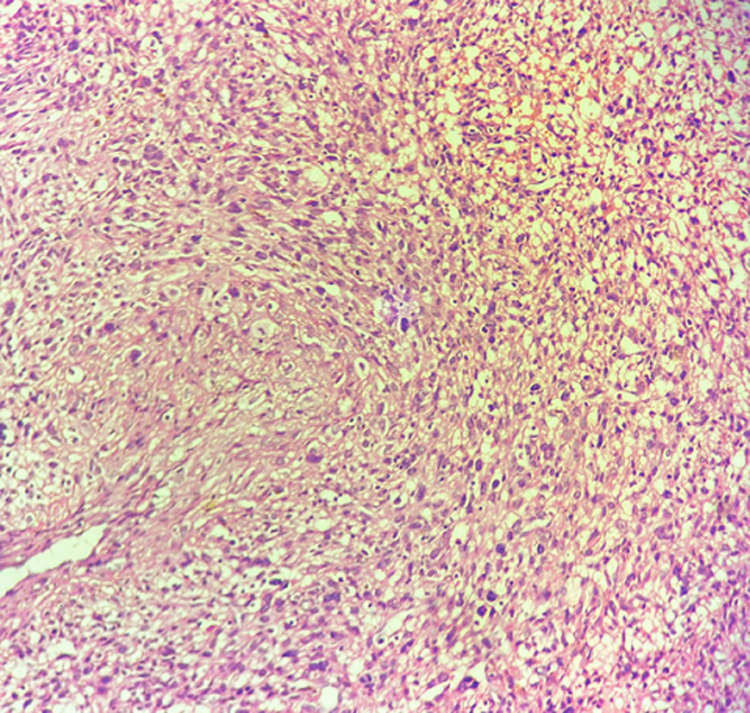
microscopic examination [H&E, 40X]- uniform population of spindle-shaped cells exhibiting slender, pleomorphic, hyperchromatic nuclei with folded contours

**Figure 5:**
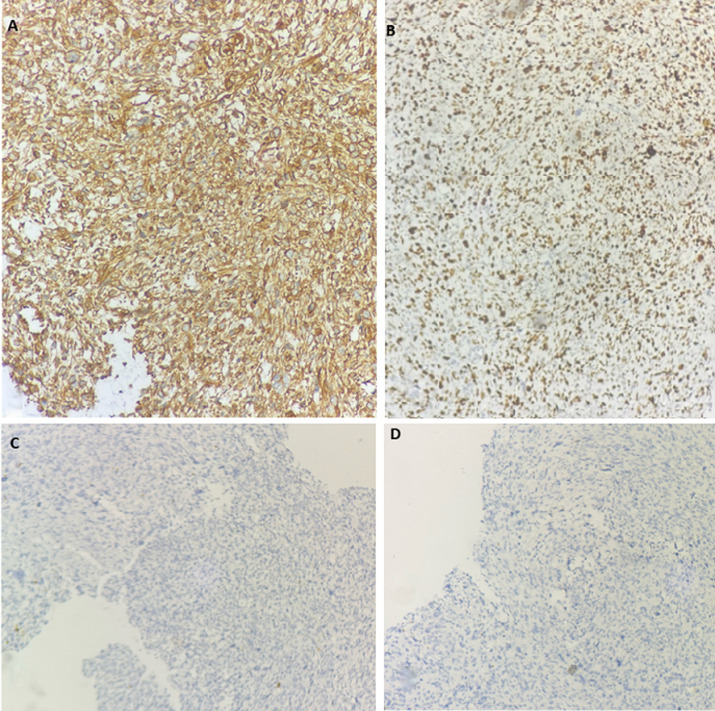
A) immunohistochemical evaluation [40x]- strong positivity for vimentin; B) immunohistochemical evaluation [20x]- markedly raised Ki-67 index, about 90%; C) immunohistochemical evaluation [10x]- negative for SOX-10; D) immunohistochemical evaluation [10x]- negative for S-100

**Diagnostic assessment:** the patient was admitted under the Department of Neurosurgery. All routine investigations, including chest X-ray and electrocardiography, were conducted and were found to be within normal limits. A previously performed MRI of the brain revealed an extra-axial lesion in the left cerebellopontine (CP) angle with a broad-based dural attachment, intense post-contrast enhancement, and a positive dural tail sign. The lesion caused indentation and distortion of the brainstem, with no evidence of vertebrobasilar abnormality, findings suggestive of a meningioma (Figure 1). Based on the clinical presentation and imaging features, a preliminary diagnosis of a left CP angle tumour was made, and the patient was scheduled for retro-mastoid craniotomy with excision of the left CP angle tumour. Intraoperatively, a well-encapsulated, greyish-white, globular mass approximating 2.4 x 1.9 cm was noted. The tumour was moderately vascular, firm in consistency, and adhered to the trigeminal nerve. The surgery was uneventful, with no trace of active bleeding or cerebrospinal fluid leakage prior to closure. The excised tissue was submitted for histopathological examination. Grossly, the specimen comprised multiple, irregular, greyish-brown fragments measuring approximately 2 x 1 x 0.5 cm (Figure 2). Microscopic evaluation demonstrated alternate hypocellular and hypercellular areas producing a marbling effect (Figure 3 A, B). Sections also showed a uniform population of spindle-shaped cells with slender, pleomorphic, hyperchromatic nuclei displaying folded contours (Figure 4). Immunohistochemical evaluation was carried out to further substantiate the diagnosis. The cells demonstrated strong positivity for vimentin (Figure 5A), a markedly raised Ki-67 index of about 90% (Figure 5B), while being negative for SOX-10 (Figure 5C) and S-100 (Figure 5D).

**Diagnosis:** histopathological and immunohistochemical assessment confirmed the diagnosis of MPNST. On microscopic evaluation, the tumour might mimic a vestibular schwannoma, which can pose further challenges in the diagnostic process. As opposed to benign CP angle lesions, MPNSTs exhibit aggressive behaviour marked by rapid growth and local invasion, eventually culminating in an unfavourable prognosis.

**Therapeutic interventions:** the patient was kept under observation in the intensive care unit for two days and, following drain removal on the second postoperative day. Thereafter, the patient was shifted to the general ward. A urology consultation was obtained, and the patient was advised to take tablet Urimax at bedtime for 15 days. He remained hemodynamically stable throughout the hospital stay and was discharged with instructions to follow up in the neurosurgery outpatient department after 10 days or earlier if any symptoms developed. The patient has been doing well on subsequent follow-up visits.

**Follow-up and outcome of interventions:** the patient was advised to attend regular follow-up visits in the neurosurgery outpatient department to monitor for any signs of recurrence or metastatic spread.

**Patient perspective:** the patient conveyed that he was pleased with the treatment and overall clinical management.

**Informed consent:** the patient provided informed consent before the preparation and publication of this case report and any associated information.

## Discussion

Malignant peripheral nerve sheath tumours (MPNSTs) are fast-growing, highly invasive soft tissue neoplasms arising from the peripheral nerve or nerve sheath cells. Intracranial MPNSTs remain exceedingly rare, where they are also known as malignant intracerebral nerve sheath tumours (MINST) [9]. Malignant peripheral nerve sheath tumours account for approximately 5-10% of all soft tissue sarcomas with an overall incidence of around 0.001%. Nearly 40-50% arise from malignant transformation in patients with NF1, while the remaining develop de novo or develop following prior radiation exposure [10]. Loss of NF1 and CDKN2A/B represents the principal genetic alterations driving the development of atypical neurofibroma (ANF). In our analysis, SMARCA2 loss was identified in 42% of cases, while no mutations or copy number variations were detected in TP53 or components of the PRC2 complex. Alongside the frequent deletion of CDKN2A/B, SMARCA2 loss was identified in 42% of ANFs; notably, in at least three cases, this deletion occurred independently of CDKN2A/B loss, indicating that disruption of the SWI/SNF (Switch/Sucrose Non-Fermentable) chromatin-remodelling complex may play a pathologic role [5].

The clinical manifestation of intracranial MPNSTs varies according to the tumour´s size and anatomical location. The predominant clinical symptoms of MPNST include pain and numbness; however, these findings are non-specific, making differentiation from other nerve lesions challenging. Despite advances in imaging and pathology, the diagnosis and management of MPNST remain difficult, and the overall prognosis is generally poor. Although rare, MPNSTs are associated with a high mortality rate, and median survival varies depending on tumour subtype and underlying molecular alterations [1]. Magnetic resonance imaging (MRI) remains the diagnostic modality of choice for MPNSTs. Lesions typically demonstrate heterogeneous signal intensity, compression of adjacent parenchymal structures, marked local invasiveness, and contrast enhancement observed in nearly all cases.

Several hypotheses have been proposed regarding the origin of MINSTs. Possible sources include Schwann cells from perivascular peripheral nerves or stromal tissue, adrenergic fibres of arterioles, meningeal branches of the trigeminal nerve, and neural crest cells that migrate abnormally during early foetal development [9].

Malignant peripheral nerve sheath tumour (MPNST) is a sarcoma-like tumour, hence it may exhibit diverse morphologic patterns on histology, which include whorls, palisades, pseudo-rosettes, and many more [2]. Typically, these tumours are composed of spindle cells arranged in an intersecting fascicle-like pattern. At low power, one can visualize hypercellular areas interspersed with hypocellular areas. In comparison to their benign counterparts, MPNSTs usually display marked hypercellularity, cellular pleomorphism, and elevated mitotic activity. These tumours also demonstrate a more organized growth pattern with minimal stromal elements [3]. This heterogeneity adds complexity to the diagnostic process, underscoring the necessity of precise histopathological diagnosis for directing proper therapeutic management. Magnetic resonance imaging (MRI) is the diagnostic modality of choice for soft tissue sarcomas. Owing to its close resemblance to other soft tissue tumours and peripheral nerve sheath tumours, both clinically and radiologically, it is challenging to tell apart MPNST from a soft tissue sarcoma [1].

The cornerstone of CP angle MPNST management is surgical resection; though complete surgical resection is often limited by the tumour´s invasiveness and its proximity to vital neurovascular structures. The overall survival rate is reported between 6 months and 2 years. According to medical literature 10-year-MPNST-specific survival rate of 31.6% has been documented, decreasing to 25.9% in recurrent cases and 7.5% in those patients with metastatic diseases. Adjuvant therapies, including chemotherapy and radiotherapy, may be considered as concomitant therapy.

## Conclusion

As a malignant soft tissue sarcoma typically arising in the trunk and limbs, MPNST is exceptionally rare in the CP angle, a location overbearingly conquered by benign lesions like vestibular schwannomas. This rarity, in combination with its highly aggressive clinical course marked by rapid progression and a proclivity to arise sporadically in the absence of the common risk factor of the NF1 gene, makes it a diagnostic challenge. Confirmation of MPNST in this location mandates a heightened sense of suspicion, as it drastically alters prognosis and management from that of presumed benign tumours.
